# Effectiveness of Multisport Play-Based Practice on Motor Coordination in Children: A Cross-Sectional Study Using the KTK Test

**DOI:** 10.3390/jfmk10020199

**Published:** 2025-05-30

**Authors:** Nicola Mancini, Rita Polito, Francesco Paolo Colecchia, Dario Colella, Giovanni Messina, Vlad Teodor Grosu, Antonietta Messina, Siria Mancini, Antonietta Monda, Maria Ruberto, Fiorenzo Moscatelli

**Affiliations:** 1 Department of Education and Sport Sciences, Pegaso Telematic University, 80143 Naples, Italy; nicola.mancini@unipegaso.it (N.M.); maria.ruberto@unipegaso.it (M.R.); 2 Department of Psychology and Health Sciences, Pegaso Telematic University, 80143 Naples, Italy; rita.polito@unipegaso.it; 3 Department of Experimental Medicine, Section of Human Physiology and Unit of Dietetics and Sports Medicine, University of Campania “Luigi Vanvitelli”, 80138 Naples, Italy; francescocolecchia@gmail.com (F.P.C.); giovanni.messina@unicampania.it (G.M.); 4 Department of Biological and Environmental Sciences and Technologies, University of Salento, 73100 Lecce, Italy; dario.colella@unisalento.it; 5 Department of Mechatronics and Machine Dynamics, Universitatea Tehnica, 400114 Cluj-Napoca, Romania; vlad.grosu@mdm.utcluj.ro; 6 Department of Precision Medicine, University of Campania “Luigi Vanvitelli”, 80138 Naples, Italy; antonietta.messina@unicampania.it; 7 Department of Humanistic Studies, University of Foggia, 71121 Foggia, Italy; siria_mancini.585707@unifg.it; 8 Department of Human Science and Promotion of Quality of Life Promotion, San Raffaele Telematic University Rome, 00166 Rome, Italy; antonietta.monda@uniroma5.it

**Keywords:** motor coordination, multisport activity, KTK, BMI, health promotion, primary school

## Abstract

**Objectives:** This study aimed to evaluate the effectiveness of a structured multisport play-based program on the development of motor coordination in Italian children aged 6 to 10 years, using the Körperkoordinationstest für Kinder (KTK). **Methods:** An observational cross-sectional design was applied, involving 320 children (mean age 8.1 ± 1.4 years; 52% male) from the Puglia region in southern Italy. Participants were divided into a Multisport Group (MG; *n* = 162), engaged in multisport activities, and a Curricular Group (CG; *n* = 158), involved in standard physical education programs. Anthropometric measurements (weight, height, and BMI) and motor coordination outcomes (Motor Quotient, MQ) were assessed using the KTK. Statistical analyses included ANOVA, Pearson’s correlations, and logistic regression models. **Results:** The results showed that the MG achieved significantly higher MQ scores (108.3 ± 12.5) compared to the CG (101.2 ± 13.4; *p* < 0.001). Moreover, higher BMI values were significantly associated with an increased risk of lower MQ scores (OR = 2.35; 95% CI: 1.45–3.81; *p* < 0.001), indicating that children with elevated BMI had more than twice the likelihood of showing impaired motor coordination. Strong positive correlations were found between all KTK subtests and the total MQ score. **Conclusions:** Participation in structured multisport programs appears to enhance motor coordination in children and may represent a valuable educational and preventive strategy within primary school physical education and public health interventions.

## 1. Introduction

Motor coordination during childhood is a crucial indicator of overall health, psychological well-being, and active participation in sports and social activities.

Adequate coordination is linked to improved academic performance, self-efficacy, and sustained physical activity engagement [1,2,3]. In Italy, there is increasing concern over a decline in motor skills among children, largely due to reduced opportunities for free play, increased sedentary lifestyles, and early sport specialization. The national “OKkio alla SALUTE” survey (ISS–Ministry of Health, 2022) [4] reports that over 45% of Italian children aged 6 to 11 fail to meet recommended physical activity levels, and 20.1% are overweight—factors associated with poorer motor development and long-term health risks. Low motor coordination is also associated with reduced physical activity, lower motor self-esteem, and elevated risk of obesity and metabolic issues during adolescence [5,6,7]. While motor coordination tends to show moderate stability over time [8], it is amenable to targeted interventions, particularly in overweight populations. Recent findings by Barnett et al. (2022) highlighted the role of multisport programs in fostering broad motor competence and inclusive participation [9]. Structured multisport interventions play a key role in reducing obesity risk and enhancing cardiovascular and neuromotor health in children [10,11,12,13,14,15]. For instance, structured programs based on rhythmic disciplines such as sport dance have been shown to improve segmental coordination and motor responsiveness in younger children, fostering holistic and multi-systemic development [16,17,18]. These programs, which emphasize variety and play, have shown to improve motor competence and promote inclusive, developmentally appropriate learning environments [19,20]. In this context, the Italian initiative “Centri CONI—Orientation and Introduction to Sport” [21] promotes an inclusive, progressive, and play-based model of motor education, offering a viable alternative to early specialization. Reliable assessment tools are essential for measuring motor coordination. The Körperkoordinationstest für Kinder (KTK) is a widely used international battery designed for children aged 5 to 14 years [22,23,24,25]. It includes four subtests (balance beam walking, lateral jumping, one-leg hopping, and sideward platform transfer) and provides a standardized Motor Quotient (MQ) adjusted for age and sex. The KTK has demonstrated strong validity in European contexts and is sensitive to training-related differences in school settings [26,27,28], although its use in Italy remains limited. This study aims to compare motor coordination levels in children from the Puglia region who participate in a structured multisport program versus those involved solely in curricular physical education. It also examines the association between BMI and KTK performance, in line with previous studies highlighting the role of body composition in motor development. Unlike earlier research, this study focuses on a large, school-based sample from Southern Italy—a region underrepresented in the literature—and evaluates the effectiveness of a structured multisport program implemented as part of the national “Centri CONI” initiative.

## 2. Materials and Methods

### 2.1. Study Design

This study employed a cross-sectional observational design, aimed at comparing motor coordination levels between children participating in a play-based multisport program and those engaging solely in standard school-based physical education. Data collection took place between May and June 2024.

### 2.2. Participants

The sample consisted of 320 children aged between 6 and 10 years (mean age = 8.1 ± 1.4 years; 52% boys). The age range (6–10 years) was selected as it corresponds to the early and middle primary school period, a critical window for motor development, during which coordination skills are particularly responsive to intervention.

Participants were divided into two groups:

- Multisport Group (MG; *n* = 162): children enrolled in the structured play-based multisport program. These children took part in a 2 h weekly program, split into two 60 min sessions (Table 1). The program was designed to develop coordinative abilities through a diverse set of motor games and sport-based activities. The project required that each child be exposed to and actively practice at least three different sports other than their primary sport of choice. Table 1 provides a monthly overview of the planned activities, including examples of specific exercises such as ball circuits, balance games, and cooperative challenges designed to progressively enhance coordination.

The sessions were conducted by sports coaches with degrees in physical education and sports sciences, all of whom had experience in childhood motor education and in the play-based multisport method. The coaches tailored the activities to the abilities and needs of each child, ensuring a stimulating and inclusive learning environment. Throughout the project, the same coaches were regularly monitored through scheduled peer-review meetings led by nationally recognized supervisors from the Italian National Olympic Committee (CONI). The Curricular Group (CG; *n* = 158) included children who only participated in standard curricular physical education, in accordance with national educational guidelines. These guidelines prescribe two hours per week of physical activity, typically focused on traditional games, basic motor exercises, and fundamental sport skills (e.g., running, jumping, throwing). The activities were conducted by generalist classroom teachers, who often had limited formal training in motor education. Inclusion criteria for both groups were age between 6 and 10 years, good general health compatible with physical activity, and regular school attendance. Children with certified motor disabilities or medical conditions preventing participation in motor tests were excluded. All procedures were approved by the Institutional Ethics Committee of Pegaso Telematic University (PROT/E 002466, 29 March 2024), and written informed consent was obtained from the legal guardians of all participants in accordance with the Declaration of Helsinki. In the Curricular Group, children who practiced any extracurricular sports activities were also excluded from the analysis.

### 2.3. Measurements

#### 2.3.1. Anthropometry

Height (in cm) and weight (in kg) were measured using standardized instruments (stadiometer and electronic scale). The measurements were taken concurrently with the administration of the KTK test, at the beginning of the evaluation period. Body mass index (BMI) was calculated as weight/height^2^ (kg/m^2^) and classified according to age- and sex-specific percentiles [29].

#### 2.3.2. Motor Coordination—KTK Test

Motor coordination was assessed using the Körperkoordinationstest für Kinder (KTK), validated for children aged 5 to 14 years. The KTK, developed by Kiphard and Schilling [30], is an internationally validated tool. It has also been adopted in several studies conducted in Italy, although a version adapted with standardized norms for the Italian population is not currently available. The test includes four motor tasks: balance beam walking (KTKBEAM), lateral jumping (KTKJUMP), one-leg hopping over obstacles (KTKHOP), and lateral movement on platforms (KTKBOARD). The same trained evaluators administered all KTK assessments throughout the study. All evaluators held university degrees in Sports and Movement Sciences and had specific experience with the KTK protocol. Prior to data collection, standardized training sessions were conducted to ensure consistent administration of the tasks and to minimize inter-rater variability. Raw scores were converted into standardized scores (MQ) based on age- and sex-specific normative values. An MQ score < 86 was considered indicative of impaired motor coordination.

### 2.4. Statistical Analysis

All statistical analyses were conducted using SPSS version 25.0 (IBM Corp., Armonk, NY, USA). In the preliminary phase, group homogeneity for age, sex, and anthropometric parameters was verified using one-way analysis of variance (ANOVA), which confirmed acceptable comparability. Outliers in BMI and MQ data were assessed through visual inspection of boxplots and standardized z-scores (cut-off ±3).

The following statistical procedures were applied:

- Descriptive statistics (mean, standard deviation, and frequencies) for all variables.
- Pearson correlation coefficients between each KTK subtest and the total MQ score.
- Eta squared (η^2^) effect sizes for between-group comparisons (Multisport vs. Curricular), interpreted according to Cohen’s criteria (η^2^ ≥ 0.01 = small; ≥0.06 = medium; ≥0.14 = large).
- Binary logistic regression to assess the association between high BMI and the likelihood of impaired motor coordination (MQ < 86).
- Multiple linear regression including interaction terms (BMI × Age, BMI × Sex, Age × Sex, BMI × Age × Sex) to explore potential combined influences on MQ. Multicollinearity was checked, and non-significant interaction terms were excluded from the final model.
- No group × age interaction was tested via ANOVA, as MQ scores are already standardized for age and sex.
- Bonferroni post hoc tests were used for exploratory comparisons between age subgroups (descriptive only).

The significance level was set at *p* < 0.05. All analyses were conducted by two independent researchers blinded to group allocation. Although the same evaluators administered the KTK, data were anonymized before analysis to reduce potential bias.

### 2.5. Statistical Power Analysis

To ensure adequate statistical sensitivity, an a priori power analysis was conducted using G*Power 3.1 [31]. Assuming a between-group difference of 7 MQ points, a standard deviation of 13, α = 0.05, and desired power = 0.80, the minimum required sample size was estimated at 150 participants per group (Cohen’s d ≈ 0.54). The final sample included 162 children in the Multisport Group (MG) and 158 in the Curricular Group (CG), thus meeting the required threshold. Additionally, a post hoc power analysis confirmed the adequacy of the sample for both the between-group comparisons and the logistic regression assessing the association between elevated BMI and impaired coordination (MQ < 86). Based on the observed odds ratios, statistical power exceeded 0.80, supporting the robustness of the findings.

## 3. Results

The data analysis revealed significant differences between the two groups in terms of motor coordination scores (MQ), as well as effects related to age and BMI.

Raw scores from each of the four KTK subtests were converted into standardized scores using official normative tables by age and sex [30,32,33]. Each subtest produces a weighted score that, when summed, yields a composite score expressed as the Motor Quotient (MQ). This score provides a comprehensive index of overall motor coordination. According to the official classification, an MQ score < 86 indicates impaired motor coordination.

To provide a more detailed overview of the participants’ motor skills, the means and standard deviations of the scores obtained in the individual KTK subtests were calculated for each group (Table 2). The MG showed higher average scores than the CG across all subtests, particularly in the Balance Beam (KTKBEAM) and Lateral Shuttle Movement (KTKBOARD) tests.

These differences suggest that the structured multisport play program may support the development of specific skills related to balance and dynamic coordination.

Table 3 reports the physical characteristics and average MQ scores of the participants, showing that the MG achieved a significantly higher mean MQ (108.3 ± 12.5) compared to the CG (101.2 ± 13.4; *p* < 0.001). Differences in physical variables (height, weight, and BMI) were minimal and not statistically significant, suggesting a good level of homogeneity between groups.

Table 4 presents the distribution of participants across the five standardized MQ categories. In the MG, 38.9% of children scored within the “good” or “very good” range (MQ > 115), compared to only 23.4% in the CG. Furthermore, no children in the MG fell into the “severely impaired” category (MQ < 70), whereas 3.2% of the CG did.

These findings suggest a protective effect of the multisport model, particularly in mitigating motor fragility and supporting children with lower initial coordination levels.

**Figure 1:**
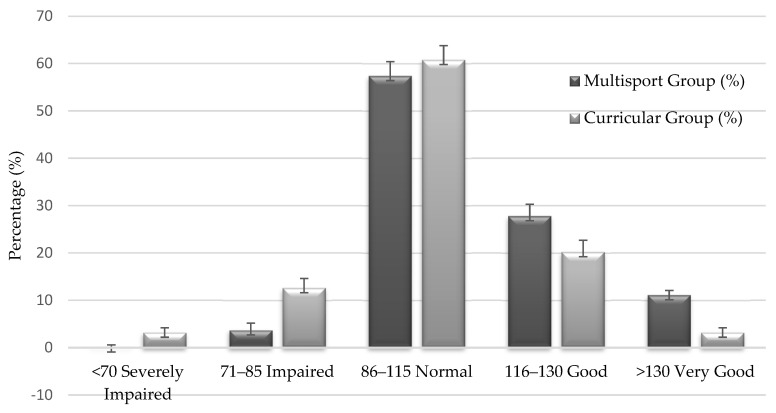
Percentage distribution of children across the different levels of motor coordination (MQ) by group (MG = Multisport Group; CG = Curricular Group), with estimated error bars (±SD). MQ categories are based on the standardized classification provided by the KTK test. This figure visualizes the MQ classification data shown in Table 4, providing a graphical representation of coordination distribution by group.

Figure 1 illustrates the distribution of MQ levels across the two groups, confirming a higher concentration of elevated scores within the Multisport Group (MG).

Table 5 shows Pearson’s correlation coefficients between the four KTK subtests and the total MQ score. All coefficients were found to be high (r ≥ 0.68, *p* < 0.001), with the strongest correlation observed in the lateral movement subtest (r = 0.76). The balance beam subtest also showed a strong correlation (r = 0.71).

These results confirm that all subtests significantly contribute to estimating overall motor coordination, and that the MQ score provides a reliable summary of the child’s motor profile. A logistic regression analysis was conducted to assess the association between high BMI and the risk of impaired motor coordination (MQ < 86).

Results showed a non-significant trend indicating a higher risk in children with elevated BMI values (OR = 1.53; 95% CI: 0.88–2.67), but the confidence interval included 1.0, suggesting a lack of statistical significance.

The multiple linear regression analysis (Table 6) revealed that the effect of BMI on MQ was significantly moderated by age (β = −0.75; *p* = 0.003). Specifically, the negative association between BMI and MQ appeared more pronounced in younger children. No significant interactions were found between BMI and sex, nor any three-way interactions among BMI, age, and sex. The post hoc power analysis for the logistic regression showed a statistical power of 0.85 to detect the effect of BMI on the risk of MQ < 86. Considering a power threshold of 0.80 as acceptable, the results suggest that the sample size was sufficient to detect a meaningful association between BMI and impaired motor coordination, thus supporting the validity of our conclusions.

## 4. Discussion

The findings of this study suggest that participation in a structured multisport play-based program is associated with significant improvements in motor coordination among school-aged children. The Multisport Group (MG) exhibited significantly higher MQ scores compared to the Curricular Group (CG), despite similar average age, weekly physical activity time, and gender distribution. These results are consistent with international literature showing that structured physical activity programs can enhance motor coordination [34,35,36,37], even in settings marked by high rates of sedentary behavior and overweight [3,8].

Previous studies have demonstrated that while motor coordination tends to be moderately stable over time (tracking), it can be improved through systematic, multicomponent, and age-adapted interventions [5,38]. Our findings reinforce this view, showing that a multisport play-based model, rich in varied stimuli, can yield tangible benefits even within a relatively short timeframe.

The strong correlations between each KTK subtest and the composite MQ score (Table 5) confirm the internal consistency of the test and support its use as a global indicator of coordination.

Moreover, the inclusivity and diversity of proposed motor tasks make this approach particularly effective in engaging children with initially lower motor skills. Although age-related trends were observed, these should be interpreted cautiously given the cross-sectional design and standardized nature of the MQ scores.

The effect sizes calculated (Table 2) indicate a medium-to-large impact of group membership (Multisport vs. Curricular) on motor coordination performance, particularly for MQ and lateral movement [8,27,28].

Another relevant finding concerns the distribution of MQ levels: The MG showed a higher incidence of scores classified as “good” or “very good” and a reduced percentage in the “impaired” category. This supports the hypothesis that a multilateral motor education approach—emphasizing variety and progression—is more effective than monothematic or unstructured models.

Regarding body composition, our results confirm that elevated BMI is associated with a higher risk of poor motor coordination. Although some findings did not reach statistical significance, the observed trends are consistent with the literature, which highlights an inverse relationship between adiposity and motor skills in children [6,39,40]. Interestingly, height appeared to be a potentially protective factor, though statistical significance was not reached in this sample.

A particularly notable result is the significant interaction between BMI and age, indicating that the negative impact of excess body weight on motor coordination (MQ) was more pronounced in younger children. This finding suggests that, during early developmental stages, higher BMI may represent a more substantial barrier to motor competence [41,42].

The regression model confirmed this interaction, supported by adequate statistical power (0.85). These results suggest that BMI should not only be considered a metabolic risk factor but also a relevant determinant of motor function and global development, especially during early school years. Moreover, school-based multisport programs may contribute to reducing disparities in motor development among children, offering inclusive opportunities for all skill levels.

### 4.1. Practical and Educational Implications

The findings of this study offer valuable insights for educators, policymakers, and health professionals, highlighting the importance of promoting structured and multisport-based physical activity programs from an early age.

- For educators and teachers, the evidence supports the adoption of didactic approaches that go beyond traditional physical education. Multilateral models emphasizing stimulus variety and progression should be prioritized. The program analyzed—comprising movement games, motor circuits, ball activities, and balance exercises—proved effective in enhancing children’s motor coordination. Integrating such models into school curricula, in collaboration with professionals in motor sciences, is highly recommended.
- For policymakers, the results underscore the effectiveness of the multisport model as both an educational and preventive tool. Investing in school- and community-based physical activity programs—coordinated by trained personnel—represents a tangible strategy for combating sedentary lifestyles, preventing childhood overweight, and fostering holistic child development. The “Centri CONI” model represents a replicable and adaptable initiative for other regions and educational systems.
- For health professionals, early motor activity plays a pivotal role in children’s psycho-physical development. Physical activity promotion should be integrated into health prevention strategies and educational campaigns targeting families. Motor coordination is not only an indicator of motor efficiency but also a predictor of active lifestyles, mental well-being, and social adaptation.
- For researchers, this study opens promising avenues for longitudinal investigations into the long-term effects of multisport programs on motor, cognitive, and socio-emotional development. Incorporating psychological and motivational variables will be essential to gain a more comprehensive understanding of the educational benefits of this approach.

### 4.2. Limitations

This study has several limitations that must be considered:

1. Cross-sectional design: This observational design limits causal inference. While associations were found, it is not possible to confirm whether the observed improvements were solely due to the program or influenced by external factors.
2. Geographic limitation: The study was conducted exclusively in the Apulia region (southern Italy) among primary school children, limiting generalizability to other regions or cultural contexts.
3. Partial control of confounders: Factors such as undeclared extracurricular physical activity, family socioeconomic status, or the quality of educational environments were not fully controlled.
4. Exclusive use of the KTK test: Although validated and widely used, the KTK assesses coordination only and does not cover other motor skills (e.g., strength, endurance). A broader assessment would provide a more comprehensive motor profile.
5. Potential bias from instructors: Despite the use of trained staff and blinded data analysis, potential observer or expectancy bias in educational settings cannot be entirely ruled out.
6. Observer variability: The KTK test requires direct observation and may be subject to inter-rater variability. However, this was minimized by using trained and experienced evaluators.

### 4.3. Future Directions

1. Longitudinal designs: needed to assess developmental trajectories and causal relationships between program participation and motor coordination.
2. Broader sample diversity: Inclusion of schools from other regions and countries would enhance generalizability.
3. Integration of psychological variables: Motivation, self-efficacy, and cognitive aspects should be included to better understand the holistic impact of multisport education.
4. Comparative effectiveness analysis: Future studies could compare the multisport model to other approaches (e.g., early specialization, free play, integrated models).
5. Combined motor and health monitoring: Future studies should integrate motor skills and health indicators (e.g., daily activity, nutrition, body composition) for a comprehensive view of child well-being.

## 5. Conclusions

This study suggests that participation in a structured, play-based multisport program—such as the Italian ‘Centri CONI’—is associated with enhanced motor coordination in school-aged children compared to traditional school physical education. Higher average MQ scores, a greater incidence of “good” and “very good” coordination levels, and fewer children in the “impaired” category within the multisport group all confirm the educational and motor benefits of the multisport model. These benefits remain significant even after adjusting for individual variables such as age, sex, and BMI. Although descriptive trends by age were observed, no inferential comparisons were conducted, and the cross-sectional design does not allow for causal inferences regarding developmental progression. This emphasizes the importance of early and sustained motor education pathways, with concurrent monitoring of coordination and anthropometric parameters. Overall, the study supports multisport-oriented physical activity as an effective tool to prevent early sedentary behavior, address motor development inequalities, and promote overall childhood well-being. However, due to the cross-sectional design, causal inferences cannot be drawn, and further longitudinal research is warranted. Based on these findings, the systematic integration of play-based multisport programs into school curricula and national educational policies is strongly recommended.

## Figures and Tables

**Table 1:** Monthly overview of activities in the Multisport Group (MG), including specific examples aimed at developing coordination through varied movement tasks.

Type of Activity	Description	Weekly Hours	Frequency	Program Duration
Movement Games	Warm-up exercises and play-based activities to improve general coordination and body awareness.	~0.4 h	2 sessions/week	8 months
Balance Exercises	Activities on different surfaces (e.g., balance beams, elastic mats) aimed at developing static and dynamic balance.	~0.4 h	2 sessions/week	8 months
Ball Activities	Ball games and drills to develop hand–eye coordination, reaction speed, and precision.	~0.4 h	2 sessions/week	8 months
Motor Circuits	Obstacle courses with varied equipment (e.g., cones, hoops, ladders) to develop agility, speed, and spatio-temporal coordination.	~0.4 h	2 sessions/week	8 months
Expressive Movement Activities	Activities including imitation, dance, and improvisation to stimulate creativity, emotional expression, and rhythmic coordination.	~0.4 h	2 sessions/week	8 months

**Table 2:** Mean scores (±SD) in the KTK subtests by group (Multisport Group [MG] vs. Curricular Group [CG]), with Eta squared (η^2^) effect size.

KTK Subtest	MG (Mean ± SD)	CG (Mean ± SD)	*p*-Value	η^2^ (Eta Squared)
Balance Beam (KTKBEAM)	29.8 ± 5.2	26.4 ± 5.9	<0.001	0.083
Lateral Jump (KTKJUMP)	58.1 ± 10.4	52.9 ± 11.2	<0.001	0.072
Single-Leg Hopping (KTKHOP)	42.5 ± 6.8	40.2 ± 7.3	<0.012	0.022
Lateral Shuttle Movement (KTKBOARD)	39.6 ± 5.9	35.1 ± 6.2	<0.001	0.091
MQ Total	108.3 ± 12.5	101.2 ± 13.4	<0.001	0.103

Note: MG = Multisport Group; CG = Curricular Group; SD = standard deviation; η^2^ = Eta squared (effect size).

**Table 3:** Physical characteristics and average Motor Quotient (MQ) scores of participants by group (MG: Multisport Group; CG: Curricular Group).

Group	N	Boys (%)	Mean Age (Years)	Height (cm)	Weight (kg)	BMI (kg/m^2^)	MQ Mean ± SD
MG	162	53%	8.0 ± 1.3	129.5 ± 6.1	26.4 ± 4.2	15.7	108.3 ± 12.5
CG	158	51%	8.1 ± 1.5	128.8 ± 6.4	27.0 ± 4.7	16.2	101.2 ± 13.4

*p* < 0.001 between groups for MQ (ANOVA); values are expressed as mean ± SD; units: years, cm, kg, and BMI in kg/m^2^. MQ = Motor Quotient.

**Table 4:** Percentage distribution of children across Motor Quotient (MQ) classification categories in the two groups: Multisport Group (MG) and Curricular Group (CG). Figure 1 also reported a visual summary of these data.

MQ Category	Description	Total (%)	MG (%)	CG (%)
<70	Severely impaired	1.6	0.0	3.2
71–85	Impaired	8.1	3.7	12.6
86–115	Normal	59.1	57.4	60.8
116–130	Good	24.1	27.8	20.2
>130	Very good	7.2	11.1	3.2

**Table 5:** Pearson correlations between KTK subtests and the total MQ score.

KTK Subtest	Correlation with Total MQ (r)
Balance Beam (KTKBEAM)	0.71 **
Lateral Jumps (KTKJUMP)	0.75 **
Single-Leg Hopping (KTKHOP)	0.68 **
Lateral Shuttle Movement (KTKBOARD)	0.76 **

Note: All correlation coefficients are statistically significant at *p* < 0.001. The MQ (Motor Quotient) represents the composite score of the KTK and summarizes the child’s overall motor coordination. ** = *p* < 0.001.

**Table 6:** Results of the multiple linear regression analysis, including coefficients (β), standard errors, t-values, and *p*-values for all predictors (BMI, age, and sex), as well as the significant interaction term (BMI × age).

Predictor	β	Standard Error	t	*p*
BMI	−2.50	0.80	−3.13	0.002
Age	3.20	0.90	3.56	<0.001
Sex (Female)	1.80	1.10	1.64	0.102
BMI × Age	−0.75	0.25	−3.00	0.003

## Data Availability

All data are include in the study.
